# An Inert Pesticide Adjuvant Synergizes Viral Pathogenicity and Mortality in Honey Bee Larvae

**DOI:** 10.1038/srep40499

**Published:** 2017-01-16

**Authors:** Julia D. Fine, Diana L. Cox-Foster, Christopher A. Mullin

**Affiliations:** 1 Department of Entomology, Center for Pollinator Research, The Pennsylvania State University, University Park, PA 16802, USA; 2 USDA-ARS-PWA Pollinating Insect Research Unit, Logan, UT 84322, USA.

## Abstract

Honey bees are highly valued for their pollination services in agricultural settings, and recent declines in managed populations have caused concern. Colony losses following a major pollination event in the United States, almond pollination, have been characterized by brood mortality with specific symptoms, followed by eventual colony loss weeks later. In this study, we demonstrate that these symptoms can be produced by chronically exposing brood to both an organosilicone surfactant adjuvant (OSS) commonly used on many agricultural crops including wine grapes, tree nuts and tree fruits and exogenous viral pathogens by simulating a horizontal transmission event. Observed synergistic mortality occurred during the larval-pupal molt. Using q-PCR techniques to measure gene expression and viral levels in larvae taken prior to observed mortality at metamorphosis, we found that exposure to OSS and exogenous virus resulted in significantly heightened Black Queen Cell Virus (BQCV) titers and lower expression of a Toll 7-like-receptor associated with autophagic viral defense (*Am18w*). These results demonstrate that organosilicone spray adjuvants that are considered biologically inert potentiate viral pathogenicity in honey bee larvae, and guidelines for OSS use may be warranted.

As generalist pollinators, honey bees (*Apis mellifera*) add an estimated 15 billion dollars of value to the crops they pollinate in the United States alone^1^. However, since 2006, managed honey bee populations have been in decline^2^,^3^. Multiple factors including pesticides, pathogens, parasitism, and poor nutrition have been implicated^4^; but at this time, no single element has been successfully shown to cause colony failure^5^,^6^. Scientists now believe that the phenomenon is a result of interacting stressors^4^,^6^,^7^,^8^. Of particular importance is the possibility of interactions between pesticides and viruses.

Honey bees are often exposed to pesticides through the residues that persist in pollen, nectar, comb wax, or on the surface of the plants in the bees’ foraging environment^9^,^10^,^11^. The social nature of a honeybee colony can result in a cascade of systemic exposures as workers forage on a contaminated food source and return to supply the other workers, queen, and developing larvae with pesticide laden food^12^. Similarly, honey bees are host to numerous picorna-like RNA viruses with multiple routes of transmission^13^,^14^. Over the foraging season, the presence and titers of these viruses vary as environmental conditions shift^15^. Additionally, foraging bees can introduce viruses to naïve hives through drift or through shared floral resources, and the resulting systemic infection can be difficult to clear^16^. Recent work has shown that pesticides can negatively impact honey bee immunity, resulting in increased viral replication^17^,^18^,^19^,^20^,^21^; however, while attention has been given to the effects and persistence of pesticide “active” ingredients, little is known about the effects of pesticide adjuvants and other ‘inerts’ in formulations.

Pesticide products are generally applied as tank mixes containing more than one formulation composed of a mixture of active and inert formulants, and adjuvants meant to enhance the penetration and spread of the active ingredients^22^. These adjuvants are generally considered by the EPA to be biologically inert; and therefore, their use is not monitored at the federal level and they are exempt from residue tolerance for food use^23^. While there is little publicly available information regarding their toxicity and residual presence in the environment, recent work has demonstrated that the residues of one class of inerts, organosilicone surfactant spray-tank adjuvant (OSS), are encountered by foraging bees and persist in honeybee food and wax^24^.

Organosilicones (Fig. 1) are a powerful class of nonionic surfactants used in agriculture as spray tank adjuvants to enhance the penetration and spread of the active ingredient^22^. Although they are classified as inerts, OSS’s have exhibited toxic effects on honeybees^25^. Worldwide OSS production in 2008 was estimated to be 1.3 billion pounds and yearly use has continued to increase^26^, while in California almonds, hundreds of thousands of pounds of OSS formulations are used every year, often during bloom^27^.

Annual California almond pollination is the largest pollination event in the United States, requiring over 60% of managed honey bee colonies in the country to be transported to the almond orchards during bloom^1^. Reports by beekeepers from 2014 and preliminary reports from 2015 describe colony die-offs characterized by healthy adults but with dying brood which are ejected from the hive as underdeveloped pupae. The hives, which face depleted populations, are at risk of failing weeks later. The use of insect growth regulators, fungicides, and adjuvants have been suggested as possible causes^28^.

Records from the California Department of Pesticide Regulation (CADPR) from 2013 report a total use of approximately 0.3 lbs. per acre of OSS adjuvants in California almonds^29^. Additional CADPR data referencing a major area of almond production, Stanislaus County, indicate that Sylgard 309 applications are commonplace during the late January to March bloom period (Fig. 2a), and that statewide organosilicone-surfactant use increased just prior to the onset of Colony Collapse Disorder and the increased colony losses observed in 2006 (Fig. 2b)^4^,^29^.

We hypothesize that the use of organosilicone adjuvants in combination with exposure to common viruses present in the hive environment and transmitted during pollination events can result in the interrupted development and brood mortality observed in hives used for almond pollination. Although there are limited data regarding the spread of diseases during almond pollination, shared floral resources and drift between colonies occurring more often due to the close proximity of the colonies^30^,^31^ creates a high-risk scenario for potential spread of viruses^16^. Furthermore, we hypothesize that exposure to OSS treatments and an immune challenge will result in an inability to clear viral infections as evidenced by higher viral loads prior to pupation.

To test these hypotheses, we have examined the effects of chronic dietary exposure of Sylgard 309, an OSS spray adjuvant blend commonly used on almonds and many other crops, on developing larvae using a sterile *in vitro* assay. Sylgard 309 is more uniform in composition than other spray adjuvants analyzed, being almost 80% pure acetyl-capped trisiloxane surfactant (Fig. 1), and it is used in spray tanks at concentrations up to 1%^32^.

Here, we report the results of an experiment that tested two factors, OSS chronic exposure and a single added viral exposure, and their interaction on the development of bees from larval to adult stage. To simulate a horizontal infectious event, the larvae were initially exposed to diet containing infectious viral material consisting of pathogens commonly found circulating in honey bee hives and foraging environments including Black Queen Cell Virus (BQCV), Deformed Wing Virus (DWV) Israeli Acute Paralysis Virus (IAPV) and Sacbrood Virus (SBV)^30^ or an equivalent volume of saline containing no infectious material. Chronic OSS exposure was assessed at 10 ppm in diet (v/v), a 1,000 fold dilution from a tank mix concentration of 1%, to simulate a potentially field relevant exposure rate^29^. After the first day, all larvae received only 10 ppm OSS or control (Ctrl) diet according to treatment group. The larvae were monitored throughout development to adult eclosion for delayed development, mortality, and symptoms of abnormal pathology. Prior to pupation, quantitative real-time PCR (q-PCR) was used to compare virus titers and gene expression between groups to determine the underlying causes of mortality seen in the treatment groups.

## Results

### Larval Rearing – Physical Symptoms

**Figure 3:**
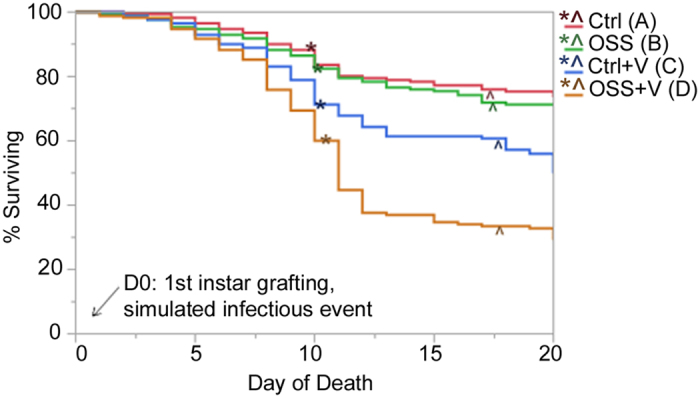
Survivorship graph of all groups. Parametric Survival significance indicated by letters, N parameters = 1, df = 1, OSS: p < 0.0034, Ctrl + V: p < 0.001, OSS + V: p < 0.0095. “*” indicates mean day of pupation. See Supplementary Fig. S1 for significance. “^” indicates mean day of adult eclosion.

**Figure 4:**
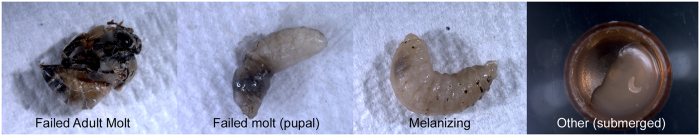
Common symptoms observed during larval rearing. Failed Adult Molt: pupae were unable to emerge from final molt successfully. Failed Molt: larvae failed to evert imaginal discs. Melanizing: Darkening internally or externally occurring in all stages. Other: No distinct symptoms could be observed, but mortality was assessed based on lack of spiracle movement, lack of growth or development for a prolonged period, or submersion in larval diet.

**Figure 5:**
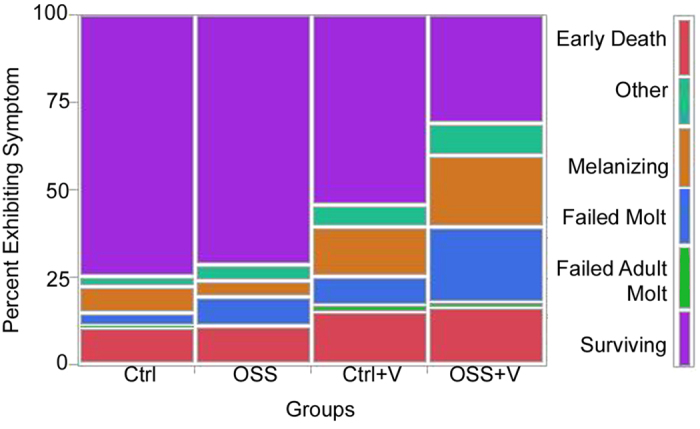
Contingency analysis graph of survivorship and observed mortality symptoms (see Table 1), grouped by treatment. N = 171, X^2^ = 30.35, df = 3, p < 0.0001.

The highest significant mortality was seen in larvae exposed to both OSS and the viral inoculum (OSS + V) (Wald test, n = 171, Wald chi square = 6.73, p ≤ 0.0095, df = 1) with over 68.4% (+/−4.32%) of the population failing before adult eclosion (Fig. 3). The highest number of individuals lost in OSS + V occurred on day 10 on the average day of pupation among individuals in this group. Many of the individuals dying during this time were observed to have attempted a molt, but died during the process with apparent melanization in the thorax and abdomen regions and lack of eversion of imaginal discs (Fig. 4). The proportion of individuals displaying this symptom at death was different between groups, (Nominal logistic regression, n = 171, X^2^ = 30.35, p < 0.0001, df = 3, Fig. 5, see Table 1 for descriptions of symptoms). Exposure to the viral inoculum was a significant predictor of this pathology (X^2^ = 12.37, p < 0.0004), and chronic exposure to OSS resulted in a positive trend toward higher numbers of individuals exhibiting this symptom (X^2^ = 3.13, p < 0.0769). Additionally, the two terms interact, resulting in a similar positive trend (X^2^ = 3.13, p < 0.0769).

In the larvae that successfully pupated, all treatment groups experienced a significant delay in pupal eclosion relative to Control larvae (Ctrl) (least squares regression, p ≤ 0.0001, df = 3, see Supplementary Fig. S1). Based on a post hoc comparison of the averages between groups, Ctrl larvae required the least time to complete development from larvae to pupae (9.8 ± 0.05 days) followed by OSS exposed larvae (OSS)(0.25 ± 0.05 day delay), larvae exposed to virus alone (Ctrl + V) (0.34 ± 0.06 day delay), and OSS + V (0.53 ± 0.07 day delay). OSS + V required a significantly longer time than Ctrl or Ctrl + V (post hoc Tukey HSD, p < 0.05); but OSS + V, OSS, and Ctrl + V were not significantly different. The delay in development was reflected in the timing of adult eclosion as determined by the appearance of wings (df = 3, p < 0.0002). Relative to Ctrl, OSS, Ctrl + V and OSS  +  V experienced a 0.15 ± 0.09, 0.43 ± 0.10 and 0.6 ± 0.14 day delay, respectively. As compared to Ctrl, the observed delays in adult eclosion were statistically significant in the treatments OSS + V and Ctrl + V (Tukey HSD, p < 0.05; but these groups were not significantly different from each other). Time to adult eclosion did not differ between the treatments OSS and Ctrl. However, the amount of time required for development between pupation and adult eclosion was not significantly different between groups, indicating that the delay in adult eclosion was directly related to delayed pupation.

### q-PCR

To test our second hypothesis, that the mortality observed in response to OSS and virus exposure together were related to elevated virus titers and compromised immunity, we examined the relative abundance of four honey bee viruses, Israeli Acute Paralysis Virus (IAPV), Black Queen Cell Virus (BQCV), Deformed Wing Virus (DWV) and Sacbrood Virus (SBV), and the expression of immune genes in larvae prior to pupation (see Supplementary Fig. S2). We explored the effects and interactions of treatments and immune gene expression on the titers of each virus. Of the viruses examined here, BQCV titers were related to three predictors: exposure to virus, exposure to OSS, and the expression of a Toll-like receptor gene (TLR) named 18-wheeler (*Am18w*)^33^ (least squares regression, n = 9–10, R^2^ adj. = 0.63, df = 7, p < 0.0001).

**Figure 6:**
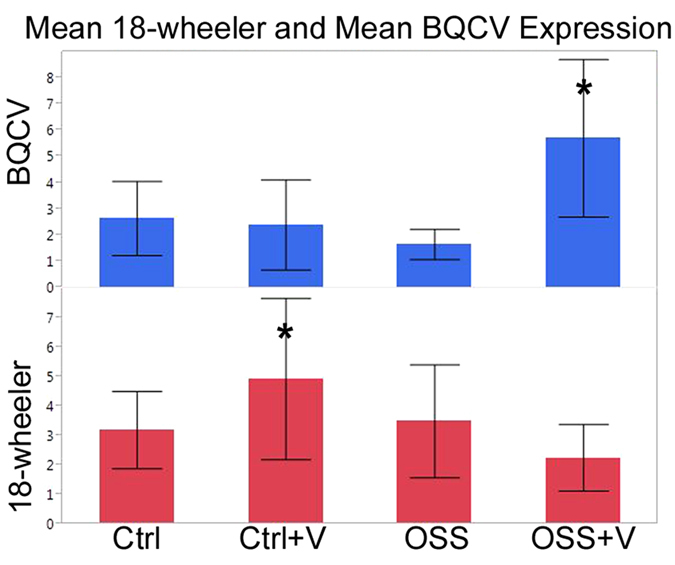
Expression or titers of 18-wheeler and BQCV according to treatment. Least squares regression, n = 9–10, R^2^ adj. = 0.63, df = 7, p < 0.0001. Standard error indicated by error bars, Response variable significance in respective models indicated by “*”. BQCV: p ≤ 0.00053, 18-wheeler: p ≤ 0.00008.

Exposure to OSS was a significant predictor of higher BQCV titers (p ≤ 0.01134) but exposure to virus alone did not result in significantly higher viral titers (p ≤ 0.08411). However, a significant interaction between exposure to OSS and viruses resulted in significantly higher BQCV titers in treatment OSS + V (p ≤ 0.00053) (Fig. 6, post hoc Tukey’s HSD, p < 0.05). The third predictor, 18-wheeler, was positively correlated with BQCV (p < 0.00001).

To further explore the three way interaction of BQCV with OSS and exposure to virus, the model was rerun with 18-wheeler as the response. Exposure to virus resulted in elevated 18-wheeler expression (p ≤ 0.00008); but, exposure to OSS depressed this expression (post hoc Student’s T-test, p ≤ 0.00001, Fig. 6). Virus exposure and OSS interacted (p ≤ 0.00001), indicating that 18-wheeler expression was significantly higher in group Ctrl + V but was lowest in OSS + V (though not significantly different than OSS or Ctrl). These results suggest that 18-wheeler expression is upregulated by exposure to viral pathogens, but depressed following exposure to OSS.

In the larvae sampled, IAPV titers correlated with BQCV titers (linear regression, n = 9–10, R^2^ adj. = 0.63, df = 3, p < 0.0001). IAPV infection levels and exposure to OSS interacted to increase BQCV titers (least squares model, n = 9–10, IAPV p ≤ 0.0001, OSS: p ≤ 0.0292, IAPV*OSS p ≤ 0.0001).

Expression of 18-wheeler was positively correlated with several other immune genes, including prophenoloxidase (least squares regression model, p ≤ 0.0001, df = 1, R^2^ adj. = 0.45), hymenoptaecin (p ≤ 0.0017, df = 1, R^2^ adj. = 0.21), and defensin 1 (p ≤ 0.0064, df = 1, R^2^ adj. = 0.16). Similar to 18-wheeler, the expression of these genes appears to be depressed in the OSS + V treated bees relative to those in bees from other treatments, but the expression levels for these immune genes are not by themselves significantly related to BQCV levels or related to treatments (see Supplementary Fig. S3). The only measured immune gene whose gene expression is significantly correlated to BQCV and treatment interactions is 18-wheeler.

## Discussion

The symptoms observed in our experiments allowed us to accurately identify patterns associated with OSS exposure and concurrent viral exposure that are most apt to be occurring during almond pollination. The high survivorship of larvae to adults in the Ctrl group allowed us to examine the impacts of chronic OSS exposure and early viral exposure (Fig. 3). OSS + V experienced a 44.45% increased mortality as compared to controls; whereas, OSS alone resulted in a 4.09% higher mortality and viral-exposure alone (Ctrl + V) had 21.56% higher mortality relative to Ctrl. If exposure to OSS and viruses had additive effects on mortality, a 25.65% loss relative to Ctrl would have been observed; however, the observed mortality with exposure to OSS and virus was nearly twice this, indicating a synergistic rather than additive interaction.

Interestingly, exposure to OSS and virus significantly affected the symptoms associated with death (Fig. 5). The deaths occurred immediately prior to the average day of pupation, and the death was most commonly characterized by a failure to evert the imaginal discs (failed molt, Fig. 4)^34^. These symptoms match those observed by beekeepers following almond pollination in 2014 and initial reports in 2016, as described by G. Wardell^28^. Hive losses were characterized by missing brood and “deformed and dying pupae”. Fungicides, insect growth regulators, and tank adjuvants applied at bloom were suggested as possible culprits.

Chronic OSS and early viral exposure during larval development were also found to affect pupal eclosion and metamorphosis (see Supplementary Fig. S1). Delayed development is a common symptom of immune challenges in insects other than honey bees^35^,^36^,^37^,^38^,^39^. The delay in pupal and adult eclosion observed in this study was related to viral infections. Insect pupation and immune function are energetically costly^37^,^40^, and immune challenges can impair growth and development^36^,^38^,^39^, perhaps resulting in the observed delay. This effect has not been previously documented in honey bees.

OSS exposure had impacts on immunity in honey bee larvae (Fig. 6). The higher BQCV titers associated with OSS treatment support our second hypothesis that OSS exposure can result in increased viral replication. Although our work did not look for active replication of the viruses, the significantly higher BQCV titers seen in OSS + V imply that replication of the virus in this group occurred at higher levels than in other groups. Almost all larvae, Ctrl or otherwise had some degree of infection, but introduction of exogenous virus in the 1^st^ instar stage resulted in significantly higher BQCV titers in OSS + V. Elevated BQCV titers were only observed when OSS exposure was combined with a simulated viral exposure event. OSS was a significant predictor of lower expression of 18-wheeler, the homolog of *Toll-7* in *Apis mellifera. Toll-7* is involved in autophagic viral defense in *Drosophila*^33^,^41^ and is expressed in the fat bodies of *Drosophila* larvae^42^. This gene may have a similar function in honey bees. Previous studies examining changes in the honey bee transcriptome induced by viral infection have not found 18-wheeler to be significantly upregulated in infected bees, but these studies largely focus on the effects of other viruses on adults and pupae^43^,^44^,^45^,^46^. This study found that 18-wheeler expression was positively correlated with viral titers in larvae prior to pupation. When larvae were subjected to viral exposure without OSS, 18-wheeler was significantly upregulated as an active immune response to viral infections.

Exposure to OSS and exogenous viruses resulted in higher BQCV titers and mortality, but the mechanisms underlying these combined impacts are not yet known. One hypothesized mechanisms is that OSS may have affected penetration of viral pathogens across the insect cuticle or peritrophic membrane. Adjuvants like OSS may enhance the penetration of pesticide active ingredients into plant tissue through several mechanisms such as by increasing penetration through the stomatal pores or by becoming embedded in the plasma membrane and altering membrane fluidity to allow direct penetration of chemicals into the cell^47^,^48^. The last of these mechanisms is poorly understood and has only been shown to occur under exposure to 100 ppm of a cationic surfactant^48^. It is theorized that this activity is dependent on the polarity of the cationic surfactant^48^. OSS’s are nonionic, and available literature has shown OSS facilitated membrane penetration to occur via the stomatal pores at high concentrations (300–1000 ppm)^49^,^50^. Further research is needed to determine whether OSS’s can alter cellular membrane fluidity and how this might affect viral infections, but the low concentration of OSS used in this study suggests a different mechanism resulted in the observed mortality. Alternatively, the increased viral titers and mortality following combined OSS and viral exposure may be due to a “perfect storm” scenario: bees exposed to viruses required activation of immune pathways such as 18-wheeler to mediate the infection but concurrent OSS exposure depressed this pathway. Transcriptomic studies have shown that pesticide exposure, poor diet, and *Varroa* parasitism can affect the expression of genes related to both nutritional regulation and immunity^51^,^52^. Although more research is needed, it may be that these stressors impact a larger pathway such as the insect TOR pathway^53^, and decreased immune function is a downstream consequence. We are currently performing experiments to examine the transcriptome of the larvae used in this study to identify pathways affected and correlated with the observed pathology.

Honey bee immune pathways engage in cross talk^54^ and the correlations observed between 18-wheeler and other immune genes may reflect this cross-talk. In *Drosophila*, a Toll activated anti-fungal peptide gene *Drosomycin* is moderately upregulated in response to viral infection but has no demonstrable role in viral defense. Other antimicrobial peptides representing the IMD and Jak-Stat pathways are not significantly affected by viral infection. In contrast, 18-wheeler has a 5 fold increase in expression in response to viral infection in *Drosophila*^41^. In honeybees, hymenoptaecin and defensin are anti-microbial peptides active against bacterial pathogens^55^,^56^, and prophenoloxidase is an enzyme precursor involved in encapsulation and melanization^57^. Although these other immune genes did not appear to be significantly affected by OSS and added viral exposure, correlative analysis suggests that they are affected indirectly by viral infection and by the activation or suppression of an immune pathway regulated by 18-wheeler (see Supplementary Fig. S3).

BQCV was the only virus studied here that was significantly affected by the treatments. Little research has reported impacts of BQCV on worker pupal development or colony survival, even though it is one of the most prevalent viruses detected in hives in the United States^15^,^58^,^59^,^60^,^61^ and its detections have significantly increased since the early 2000’s. In this work, the synergistic increase in mortality during development observed after treatment with OSS plus viral inoculum was associated with increased BQCV titers. BQCV is typically associated with death in the prepupal stage^58^,^62^, which was also when the highest mortality was observed in this study. In contrast, SBV, which was not affected by treatment and was present only at low levels in the inoculum, produces death prior to pupation; the symptoms produced by SBV^63^ are inconsistent with the symptoms observed in this study. In brood, BQCV infection is thought to occur through horizontal transmission from infected nurse bees or though vertical transmission from infected queens^64^,^65^. The virus, which replicates in worker and queen brood^62^,^65^, causes death in developing bees^62^,^65^,^66^,^67^, and although the majority of research has focused on its effects on developing queens, it has been shown that oral exposure to BQCV at high levels (10^9^ genome equivalents) and the neonicotinoid pesticide thiacloprid results in additive larval mortality and an increase in BQCV titers^21^.

The relationship between BQCV and IAPV suggests that the co-infection of IAPV with BQCV may enhance BQCV infections. Likewise, the predictive relationship between IAPV titers and OSS exposure on BQCV suggest that the IAPV in the virus inoculum may have influenced the virulence of BQCV in larvae that had been immuno-compromised via OSS exposure. Recent work reveals that exposure to different virus combinations results in different viral dynamics within the honey bee host^68^, and co-infections are often a predictor of colony death^69^. There is evidence that IAPV encodes an immune-suppressive protein and that the virus affects energy-related processes. Both of these effects could have consequences for the overall immune function of the host^46^,^70^, perhaps resulting in the worsening of a co-infection. Indeed, IAPV infections have been suggested as a potential predictor of colony failure^71^ that may occur because of this putative immune-suppressive activity. This suggests that the virus may facilitate opportunistic infections. Therefore, we speculate that interactions between introduced and exogenous viruses may also have influenced the observed titers. Future studies involving cell lines and BQCV and IAPV isolates could elucidate this relationship.

The parallels between the symptoms observed in hives following almond pollination and the symptoms observed in this study are striking and support the hypothesis that OSS use in combination with other stressors can lead to the brood mortality observed following almond pollination. A recent survey of virus prevalence in hives before, during and after almond pollination found that the highest abundance of viral pathogens occurs following almond pollination, implying that infections are spread or worsened during the event^54^. Of the viruses examined in this study, BQCV was one of the most commonly detected viruses in colonies used for almond pollination. Currently, hundreds of thousands of pounds of organosilicone adjuvant are used every year in California almond orchards alone^26^. Sylgard 309 is one of the top three out of 45 different OSS products used on almonds while bees are pollinating^29^; and though the issue is complicated by diversity of products and spray mix combinations, almond orchards are a major crop site where monitoring of OSS use should be considered. OSS’s are widely used in pesticide tank mixes applied to agricultural crops including wine grapes, tree nuts and tree fruits^26^, and there are currently no restrictions on their use around pollinating insects^22^,^23^. Potentially, other beneficial insects may experience similar symptoms in response to OSS exposure, and the continued decline of pollinator populations suggests that OSS use be evaluated. Residue studies to assess the persistence of OSS in the environment should be considered to further characterize the risk of OSS adjuvants. Furthermore, many other agrochemical adjuvant classes and inert formulants that are currently unmonitored and understudied could have similar consequences, and evaluating their safety may be warranted.

## Methods

### Honey Bee Colonies

Three original colonies of *Apis mellifera ligustica* (hives 1, 2, and 3) and two splits (derived from hive 2 and 3) from a Pennsylvania State University apiary (University Park, PA 16802) were selected based on relative health and surveyed for the presence of viruses. To ensure uniform age among larvae, the queen was caged on a frame for 24 hours and then excluded from the frame until it was collected for grafting three days later. The experiments commenced in June 2015, and the last frame was taken in late July 2015.

### Chemicals and Virus Inoculum

Sylgard 309, lot no. 0006018786 was obtained from the Dow Corning Corp., Midland, MI, USA. It is not currently possible to obtain purified strains of honey bee viruses, therefore, a semi-purified virus solution was prepared using a protocol described in Singh 2010 *et al*.^30^. Five adult bees (collected from colonies exhibiting symptoms of IAPV) were homogenized in 5 µL saline solution, and the solution was centrifuged to remove large debris and further purified using a 0.2 micron filter.

As measured using a Primerdesign^TM^ Genesig kit for the IAPV genome, the solution contained 940 genome equivalents of IAPV/μL of inoculum (see Supplementary Fig. S4). However, the virus inoculum was composed of four known viruses and relative quantification data for the inoculum showed that IAPV was amplified roughly six cycles after both BQCV and DWV. SBV was amplified roughly four cycles following IAPV. Comparable parameters including similar melting temperatures and similar primer and amplicon sizes were used for all viruses; therefore, the differences in amplification times are likely to reflect the relative abundance of each virus.

### Rearing

The larvae were grafted and reared using a modified version of a protocol described in Schmehl *et al*.^72^. This protocol, based on the rearing protocol developed by Crailsheim *et al*.^73^, uses sterile technique to minimize the unintended exposure of the larvae to pathogens, and ensures stable and clean conditions during development. We modified the protocol by performing the grafting and feedings in a specialized sterile and heated box utilizing a commercial HEPA air filter fitted to vacuum tubing to generate sterile air flow and maintain positive air pressure in the chamber and a temperature/solid state relay to control the heating output of two 350-watt heating elements wired in parallel by referencing output air temperature (see Supplementary Fig. S5). Exposure temperature of larvae was tightly controlled by use of an incubator or warm boxes to hold plates during observations.

### Experimental Design

Within 24 hours of hatching, four plates of 24 larvae each were grafted onto larval diet. Two of these plates contained diet spiked with 10 ppm(v/v) Sylgard 309 (OSS) and two with an equivalent volume of Milli-Q purified water (Ctrl). This concentration was selected following an oral communication with an industry representative suggesting a thousand fold dilution from the tank mix concentration of 1% as a field realistic dose of OSS. To one of the Ctrl plates (Ctrl + V) and one of the OSS plates (OSS + V), virus inoculum (approx. 47 copies of virus per larva) was added as a one-time exposure. An equivalent volume of saline was added to the Ctrl and OSS diets. Additionally, a positive control treatment group was present in each experimental replicate. See Supplementary Methods for details. Each treatment group was transferred to an incubator following grafting, and isolated from other groups in a humidity controlled desiccator. During larval development, OSS and OSS + V groups were fed a diet of 10 ppm OSS, and groups Ctrl and Ctrl + V were fed a diet containing an equivalent volume of Milli-Q water. Mortality and associated symptoms as well as developmental landmarks were monitored and recorded daily while carefully keeping the larvae warm. This experiment was repeated three times each for each of the three hives. In the event of queen loss, a split of the original hive was used for the remaining 1-2 repetitions (see supplementary methods for more details).

Immature bees were photographed upon death and visible symptoms (Fig. 4) and date post-graft were recorded.

### q-PCR

On day six of the larval rearing, 24 hours following the last scheduled feeding and immediately prior to transferring larvae to pupation plates, five larvae were removed from each plate and frozen at −80 °C for future RNA extraction. Larvae selected were healthy in appearance and had consumed all of the provided diet. q-PCR was performed using primers for IAPV, BQCV, DWV, SBV and immune and detoxification genes, including 18-wheeler, hymenoptaecin, defensin-1, and prophenoloxidase (see Supplementary Table S6). Novel primers were verified by Sanger sequencing of cloned products. Three experimental replicates, one from each bio-replicate, were selected for analysis (see supplemental materials for details).

### Statistical Analysis

Survivorship analysis (survival analysis and parametric regression), standard least squares regression, and contingency analysis were performed and graphs were prepared using JMP Version Pro 12. Significance was evaluated using a 0.05 probability threshold. Survivorship analysis was performed across all hives and reps (n = 171), and Wald tests were used to evaluate differences. Tukey’s HSD test or Student’s T-test were used to compare least squared means of response variables between groups. Nominal logistic regression was evaluated using a likelihood ratio test. All symptoms are displayed in Fig. 4, but symptoms were simplified into two categories, failed molt and other, for the analysis reported. See Supplementary Methods for additional details.

## Additional Information

**How to cite this article:** Fine, J. D. *et al*. An Inert Pesticide Adjuvant Synergizes Viral Pathogenicity and Mortality in Honey Bee Larvae. *Sci. Rep.* **7**, 40499; doi: 10.1038/srep40499 (2017).

**Publisher's note:** Springer Nature remains neutral with regard to jurisdictional claims in published maps and institutional affiliations.

## Supplementary Material

Supplementary Material

## Figures and Tables

**Figure 1:**
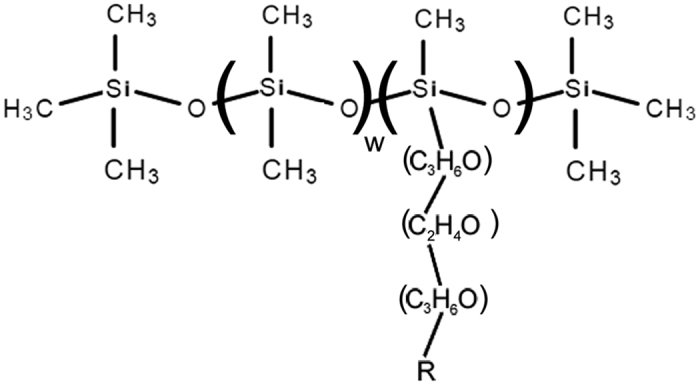
General organosilicone surfactant structure. For more details, see reference 24. Sylgard 309 is primarily R = acetyl, w = 0 with a variable polyethoxylate tail.

**Figure 2:**
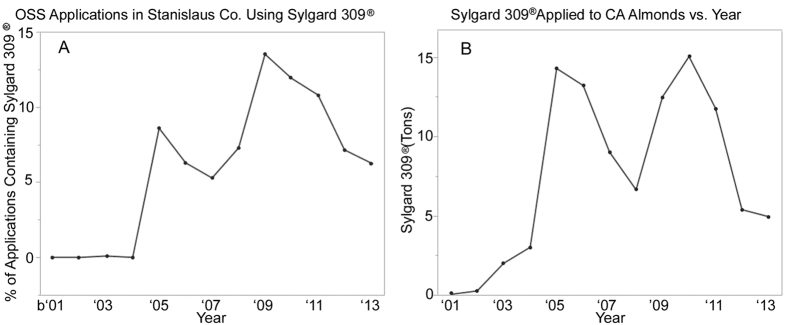
(**a)** (Left) The percentage of OSS containing applications using Sylgard 309 in Stanislaus Co. from 2001 to 2013. (**b)** (Right) Tons of OSS used on California almonds from 2001 to 2013.

**Table 1:** List and description of symptoms observed in larvae and used in contingency analysis in Fig. 5.

Symptom	Description
Surviving	Survived and successfully eclosed as an adult bee
Other	No apparent symptoms other than arrested development and sometimes discoloration
Melanizing	Appearance of dark nodules or all over darkening of larvae not related to cuticle hardening
Failed molt	Appearance of a head capsule or other pupal characteristics, but the pupal molt is incomplete. May present with melanized nodules forming.
Failed adult molt	Failure to emerge from final molt
Early death	Submersion in diet, flattening, lack of spiracle movement or melanizing occurring prior to transfer to pupation plate
