# Supplementary material for: An Inert Pesticide Adjuvant Synergizes Viral Pathogenicity and Mortality in Honey Bee Larvae
Source: Sci Rep. 2017 Jan 16;7:40499. doi: 10.1038/srep40499 (PMC5238421; doi:10.1038/srep40499)
Supplement: Supplementary Material [file srep40499-s1.pdf]

An Inert Pesticide Adjuvant Synergizes Viral Pathogenicity and Mortality in Honey Bee Larvae

Julia D. Fine[1*], Diana L. Cox-Foster[2], Christopher A. Mullin[1]
[1]Department of Entomology, Center for Pollinator Research. The Pennsylvania State University, University Park, PA 16802
[2]USDA-ARS-PWA Pollinating Insect Research Unit, Logan, UT 84322
[*]jdf250@psu.edu

Supplementary Methods

Honey Bee Larval Rearing. Hives used in this experiment were selected based on low prevalence of viruses in nurse bees (five from each of 5 hives, total N=25) taken within 2 weeks of the first grafting from each hive. All hives and the virus inoculum were negative for SBV. IAPV was detected in one individual in hive 1, BQCV was detected in one individual in a hive 2 split, and DWV was detected in 3 individuals in the hive 2 split. Based on the relatively low levels of the viruses of interest detected in this screening, these hives were deemed suitable for this work.

Larvae were reared according to a modified protocol described in Schmehl et al. 2016[1]. Because a sterile environment is critical when performing the initial grafting and subsequent feedings, a sterile, temperature controlled cabinet was designed and assembled by Randall A. McCullough, Senior Research Aide in Noll Laboratories at Pennsylvania State University. The 91.5 cm wide, 61 cm tall and 61 cm deep frame was assembled using 80/20® Quick Tube material and covered in Frost King® shrink film window insulation. A Plexiglass sash with adjustable height served as the opening through which grafting and feeding manipulations were performed. The chamber was placed on an aluminum covered surface and was sterilized with ethanol and a bleach solution before and after each use. To ensure sterile air flow throughout the chamber, a commercial HEPA air filter was fitted to vacuum tubing used to generate positive air pressure in the cabinet. To control the heat of the filtered air, a temperature/solid state relay was used to control the heating output of two 350 watt heating elements wired in parallel by referencing output air temperature. An image of the chamber is shown in Fig. S5. Aside from the use of the sterile chamber, the protocol described in Schmehl et al. 2016[1] was followed.

Grafting commenced on June 1, 2015 and the final round was performed on July 5, 2015. Fig. S7 depicts the arrangements and relationships of the hives used in this study and the exact dates that each grafting was performed. Due to the unpredictable nature of honeybee hives as a model organism, splits of the original hiveswith sister queens were used as replacement colonies in the event of queen death or absconding. Colonies 2 and 3 both experienced queen death during the course of the experiment, and a sister colony (split from the original colony with a

daughter queen) was used to complete the remaining replicates. While we acknowledge that this is an imperfect replication, we believe this solution to be the most practical and effective. Total control of the genetic heritage of the brood used in these experiments is beyond the scope of this research and may not be of practical importance for beekeepers.

Positive Control. Because the toxicity of OSS to larvae was unknown and the mode of action has not been previously explored, we did not use a traditional positive pesticide control such as dimethoate. Instead, we administered a chronic dose of OSS at 100 ppm v/v to larvae in diet (OSS-100). Based on log-rank significance tests, the survivorship of OSS-100 to adult eclosion was significantly lower than Ctrl or OSS (10 ppm), higher than OSS+V(10 ppm) but not significantly different than Ctrl+V ($p \leq 0.000$, 0.002, 0.002, and 0.9472 respectively).

RNA Extraction. In the interest of time and limited money, RNA was extracted from the first experimental replicate in hive 1, the second from hive 2, and the third from hive 3 (second from hive 3-split). These selections were made in an effort to consider variations in time and space. Samples were further selected following the extractions based on the concentration and purity of RNA. Individual larvae were manually homogenized using a plastic pestle and RNA extraction was carried out using TRIzol® Reagent (Invitrogen, Carlsbad, CA) according to the manufacturer's method. Homogenized larvae were vortexed with 0.5 mL Trizol and incubated for 3 minutes at room temperature before centrifugation. Precipitated RNA was purified using the RNA Clean and Concentrator™-5 kit (Zymo Research, Irvine, CA). Samples were selected based on the purity of extraction (260 nm/280 nm absorbance ratios above 1.8) Ctrl samples were comprised of 3 larvae from hive 1, 2 from hive 2, and 4 from hive 3 for a total N=9. Ctrl+V samples were comprised of 3 larvae from hive 1, 3 from hive 2, and 4 from hive 3 for a total N=10. OSS samples were comprised of 3 larvae from hive 1, 3 from hive 2, and 4 from hive 3 for a total N=10. OSS+V samples were comprised of 3 larvae from hive 1, 3 from hive 2, and 3 from hive 3 for a total of N=9.**RT-PCR.** CDNA was synthesized using M-MLV Reverse Transcriptase (Promega Corporation, Madison, WI) using the protocol described in Cox-Foster et al. 2007[2]. RT-PCR was performed using Sybr® Green Master Mix (Life Technologies, Carlsbad, CA) according to the manufacturer's protocol. For actin, IAPV, DWV, and SBV, the following thermal profile was used: 8 minutes at 94 °C, 35 cycles of 1 minute at 94 °C, 55 seconds at 51.5 °C, and 1 minute 25 seconds at 72° C, and an extension step of 10 minute at 72 °C. For BQCV, the thermal profile used was as follows: 8 minutes at 94 °C, 38 cycles of 1 minute at 94 °C, 1 minute at 55 °C, and 1 minute 15 seconds at 72 °C, and an extension step of 10 minute at 72 °C.

qRT-PCR. Q-RT-PCR was performed using Power Sybr® Green Master Mix (Life Technologies, Carlsbad, CA) with a reaction volume of 20 µL composed of 10 µL master mix, 5 µL nuclease free water, 2 µL each of forward and reverse primers, and 1 µL cDNA template. The following thermal profile was used: 2 minutes at 50 °C, 10 minutes at 95 °C, 40 reps of 15 seconds at 95 °C and 1 minute at 60 °C, and a dissociation step of 15 seconds at 95 °C, 1 minute at 60 °C, and 15 seconds at 95 °C. The temperature at which each product dissociated was used

to ensure that the correct product was amplifying. To verify the novel primer set used to quantify the expression of 18-wheeler, a new reverse primer was used to generate a longer product containing the original product used for q-PCR. Following gel purification, The product of this primer set was validated by Sanger sequencing of the subcloned purified product (pGEM®T Vector System, Promega Corporation, Madison, WI). All PCR primers are listed in Supplementary Table S6.

Data Analysis. All expression and virus data were normalized to actin, an established and reliable endogenous control, and expression was calculated according to the formula $2^{-\Delta Ct}$. The ΔCt was calculated by subtracting the Ct (or cycle time at which a fluorescent signal is amplified above a threshold) of actin from the Ct of the gene or virus of interest. The ΔCt method was used instead of the $\Delta\Delta$Ct method, which compares the ΔCt of a treatment group to a control group, in order to facilitate comparisons of individual data points[3]. The number of samples used in each group are as follows: Ctrl n=9, OSS n=10, Ctrl+V n=10, OSS+V n=10.

Absolute quantification of IAPV in the virus inoculum was performed using Microsoft Excel 2010®. The calibration curve used is shown in Fig. S4. Statistical analysis was accomplished using JMP Pro 12® (SAS Institute, Cary, NC).

Regression analysis was performed to determine whether seasonality affected the outcome of the experiment, using day of grafting to predict day of death. No significant relationship was found (n=168, R^2 adj.=0.000215, df=682, p<0.2846). To identify hive effects, a least squares regression model was fit to each gene or virus of interest in response to hive source. IAPV and BQCV titers and 18 wheeler expression were significantly predicted by hive (least squares regression, hive 1 and 2: n=12, hive 3: n=15; IAPV: R^2 adj.=0.290, df=38, p<0.0008; BQCV: R^2 adj.=0.113, df=38, p<0.0433; 18-wheeler: R^2 adj.=0.298, df=38, p<0.0006). According to a post hoc Tukey's HSD test, the highest titers of IAPV and the highest expression of 18-wheeler were observed in hive 2. Titers of BQCV were found to be significantly higher in hive 2 compared to hive 1, and titers in hive three were intermediate according to a Tukey's HSD test. . When hive was used to predict the day of death of the larvae using a standard least squares model, individuals in hives 2 and 3 were found to die significantly sooner than individuals in hive 1. This data demonstrated that viral titers and mortality varied between individuals from different hives in a similar manner. The increased expression of 18-wheeler and the higher IAPV titers in hive 2 suggests that this gene is an important player in defense against these viruses.

Supplementary References

1. Schmehl, D. R., Tomé, H. V. V., Mortensen, A. N., Martins, G. F. & Ellis, J. D. Protocol for the *in vitro* rearing of *Apis mellifera* workers. *J. Apic. Res.* **55,** 113–129 (2016).
2. Cox-Foster D. L. *et al.* A metagenomic survey of microbes in honey bee colony collapse disorder. *Science* **318**, 283–287 (2007).

3. Schmittgen, T. D. & Livak, K. J. Analyzing real-time PCR data by the comparative CT method. *Nat. Protocols* **3**, 1101–1108 (2008).
4. Shen, M., Cui, L., Ostiguy, N. & Cox-Foster, D. L. Intricate transmission routes and interactions between picorna-like viruses (Kashmir bee virus and sacbrood virus) with the honeybee host and the parasitic varroa mite. *J. Gen. Virol.* **86**, 2281–2289 (2005).
5. Singh, R. *et al.* RNA viruses in hymenopteran pollinators: evidence of inter-taxa virus transmission via pollen and potential impact on non-*Apis* hymenopteran ppecies. *PLoS ONE* **5,** e14357 (2010).
6. Shutler, D. *et al.* Honey bee *Apis mellifera* parasites in the absence of *Nosema ceranae* fungi and *Varroa destructor* mites. *PLoS ONE* **9**, e98599 (2014).
7. Chantawannakul, P., Ward, L., Boonham, N. & Brown, M. A scientific note on the detection of honeybee viruses using real-time {PCR} (TaqMan) in Varroa mites collected from a Thai honeybee (Apis mellifera) apiary. *J. Invert. Pathol.* **91,** 69–73 (2006).
8. Yang X. & Cox-Foster D.L. Impact of an ectoparasite on the immunity and pathology of an invertebrate: Evidence for host immunosuppression and viral amplification. *Proc. Natl. Acad. Sci. USA* **102**, 7470–7475 (2005).
9. Evans, J. D. *et al.* Immune pathways and defence mechanisms in honey bees Apis mellifera. *Insect Molec. Biol.* **15,** 645–656 (2006).

Supplementary Figures

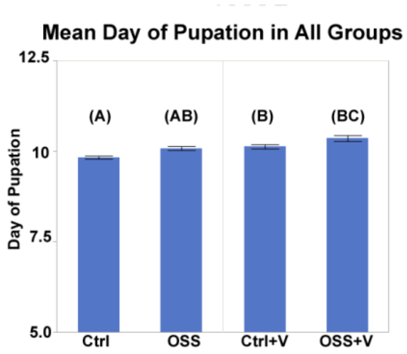

Supplementary Figure S1. Mean day of pupation after hatching for larvae following treatment (Control (Ctrl), OSS treated larvae (OSS), Added viral exposure (Ctrl+V), and OSS treatment/Added viral exposure (OSS+V)). (Least squares regression, $p \leq 0.0001$, df=3. Tukey HSD significance indicated by letters.

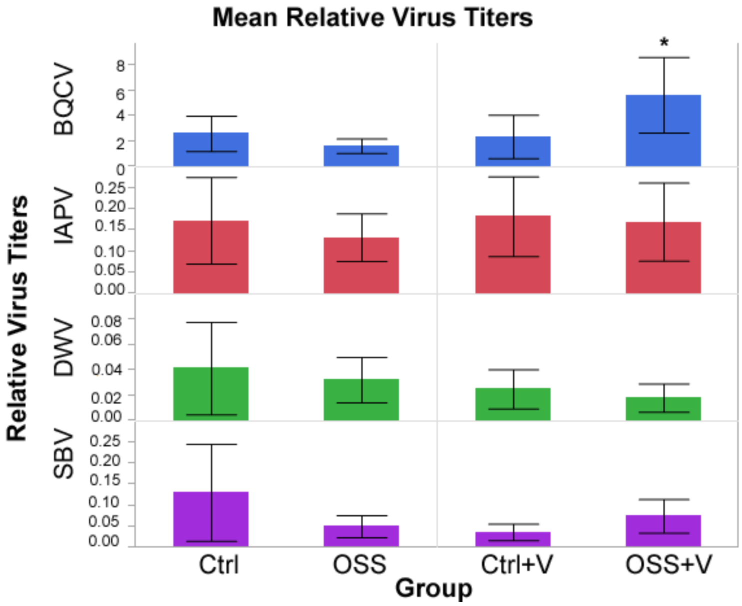

Supplementary Figure S2. Average relative titers for Black Queen Cell Virus (BQCV), Israeli Acute Paralysis Virus (IAPV), Deformed Wing Virus (DWV), and Sacbrood Virus (SBV) for all treatment groups (Control (Ctrl), OSS treated larvae (OSS), Added viral exposure (Ctrl+V), and OSS treatment/Added viral exposure (OSS+V)). Viral titers in each bee were normalized to beta-actin levels in that bee. (Significance tested using least squares regression, n=9-10. Model using BQCV: R^2 adj.=0.63, df=7, $p<0.0001$). Tukey HSD significance indicated by "*".

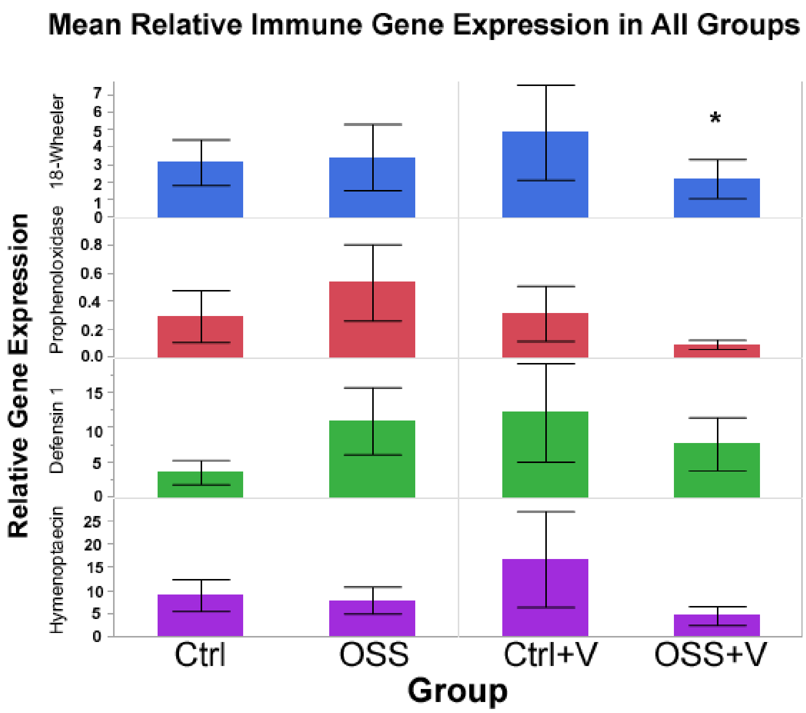

Supplementary Figure S3. Relative expression of immune genes in day 6 larvae following treatment (Control (Ctrl), OSS treated larvae (OSS), Added viral exposure (Ctrl+V), and OSS treatment/Added viral exposure (OSS+V)). Tukey HSD significance indicated by "*".

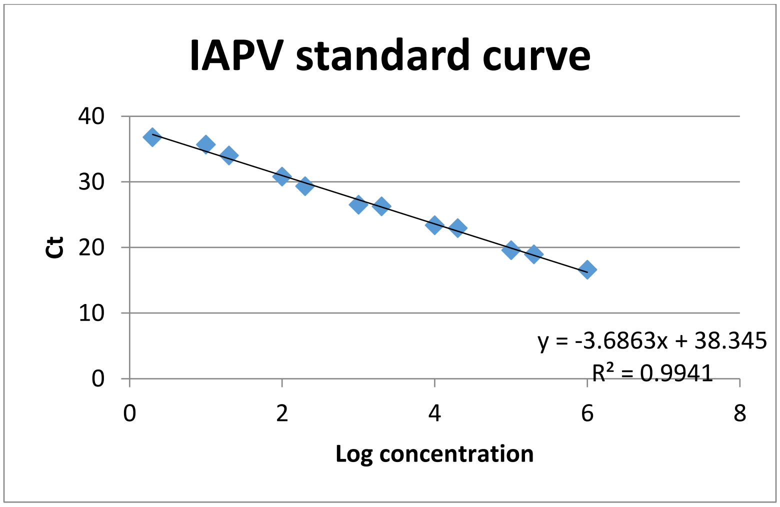

Supplementary Figure S4. Absolute quantification curve for Israeli Acute Paralysis Virus (IAPV), used to determine the IAPV in viral inoculum. Ct = cycle time, Log concentration = log of copy number in q-PCR reaction.

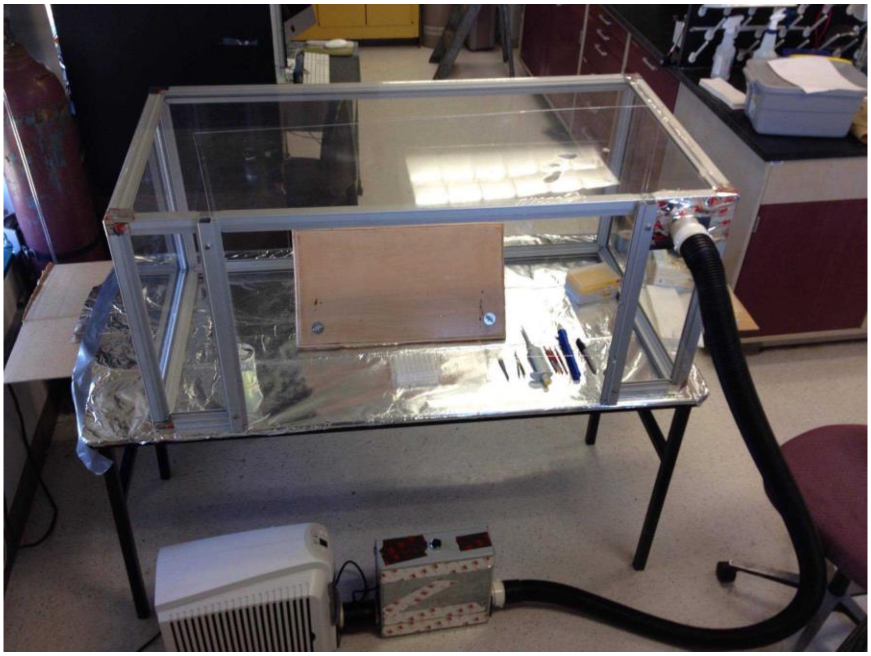

Supplementary Figure S5: An image of the sterile chamber used for grafting, feeding and mortality assessments.

Supplementary Table S6. All PCR primers used in this study.

**Rt-PCR Primers**				
**Gene/Virus**	**Primer sequence**	**Product (bp)**	**Accession no.**	**Source**
Actin Forward	ATGAAGATCCTTACAGAAAG	514	BI504901	Shen 2005[4]
Actin Reverse	TCTTGTTTAGAGATCCACAT			
IAPV Forward	GGTCCAAACCTCGAAATCAA	840	NC009025	Singh 2010[5]
IAPV Reverse	TTGGTCCGGATGTTAATGGT			
DWV Forward	CTCGTCATTTTGTCCCGACT	424	NC004830	Singh 2010[5]
DWV Reverse	TGCAAAGATGCTGTCAAACC			
BQCV Forward	TGGCAACCTAGCCATTTAGC	792	NC003784	Shutler 2014[6]
BQCV Reverse	GGTAGTGGGAGCTGACCAAA			
SBV Forward	CACTCAACTTACACAAAAAC	210	AF092924	Shen 2005[4]
SBV Reverse	CATTAACTACTCTCACTTTC			
**Q-Rt-PCR Primers**				
**Gene/Virus**	**Forward Primer**	**Product (bp)**	**Accession no.**	**Source**
IAPV Forward	TGAACCAGGAGTGATACCCGTTA	840	NC009025	Singh 2010[5]
IAPV Reverse	CGCCGTTCCTGTGAGTTGAT			
DWV Forward	CTCGTCATTTTGTCCCGACT	63	NC004830.2	Shutler 2014[6]
DWV Reverse	TTCATCAGGAGCACAACCTACAG			
BQCV Forward	GGTGCGGGAGATGATATGGA	71	NC003784.1	Chantawannakul 2006[7]
BQCV Reverse	GCCGTGTGAGATGCATGAATAC			
SBV Forward	TGATGCAGTAGCTATGCGAGTGA	77	NC002066.1	Levitt, Cox-Foster, unpublihed
SBV Reverse	GCGGCTCATCAGGATAAGCA			
Hymenoptaecin Forward	ATATCCCGACTCGTTTCCGA	201	NM001011615.1	Yang 2005[8]
Hymenoptaecin Reverse	TCCCAAACTCGAATCCTGCA			
PPOact Forward	GTTTGGTCGACGGAAGAAAA	274	XM001121888.3	Evans 2006[9]
PPOact Reverse	CCGTCGACTCGAAATCGTAT			
Defensin Forward	TGCGCTGCTAACTGTCTCAG	119	NM001011616.2	Evans 2006[9]
Defensin Reverse	AATGGCACTTAACCGAAACG			
18-wheeler Forward	CGCTGATCAACGCAGCTTTT	73	NM_001013361.1	this work
18-wheeler Reverse	CACTTGCAAGACGTTCCAGC			
18-wheeler long Forward	CGCTGATCAACGCAGCTTTT	457	NM_001013361.1	this work
18-wheeler long Reverse	GCTTGCACATTGCTCTCCAC			

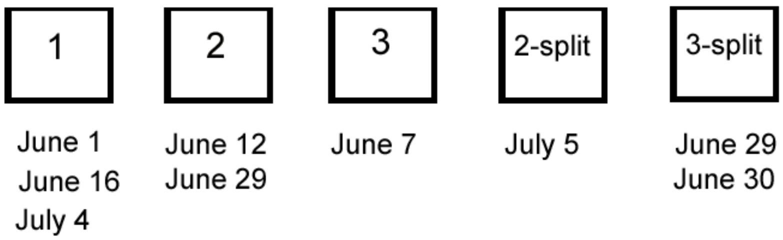

Supplementary Figure S7. Arrangement of hives and dates of grafting used in this study.
